# Occurrence and Risk Factors of Brucellosis in Commercial Cattle Farms from Selected Districts of the Eastern Coast Zone, Tanzania

**DOI:** 10.1155/2023/4904931

**Published:** 2023-02-13

**Authors:** James P. Warioba, Esron D. Karimuribo, Erick V. G. Komba, Mwemezi L. Kabululu, Godwin A. Minga, Hezron E. Nonga

**Affiliations:** ^1^Zonal Veterinary Center, Ministry of Livestock and Fisheries, P.O. Box 1068, Arusha, Tanzania; ^2^Department of Veterinary Medicine and Public Health, College of Veterinary Medicine and Biomedical Sciences, Sokoine University of Agriculture, P.O. Box 3021, Chuo Kikuu, Morogoro, Tanzania; ^3^Tanzania Livestock Research Institute (TALIRI) - Central Zone, P.O. Box 202, Mpwapwa, Dodoma, Tanzania; ^4^Tanzania Veterinary Laboratory Agency, Central Veterinary Laboratory Ministry of Livestock and Fisheries, 131 Mandela Road, P.O. Box 9254, Dar Es Salaam, Tanzania; ^5^Department of Veterinary Services, Ministry of Livestock and Fisheries, P.O. Box 2870, 47480, Dodoma, Tanzania

## Abstract

Brucellosis is a disease of major socio-economic importance worldwide, particularly in low-income countries. This retrospective study aimed to estimate seroprevalence and risk factors associated with brucellosis in commercial cattle farms in the eastern coast zone of Tanzania (ECZT). A total of 1,052 serum samples collected from 20 commercial farms were subjected to rose bengal plate test (RBPT) and indirect enzyme-linked immunosorbent assay (i-ELISA). Descriptive analysis was employed to determine frequencies and proportions. To establish risk factors, a multivariate logistic regression analysis was carried out using a backward elimination procedure, following a univariate analysis, with 0.1 set as a cut-off point for the selection of putative risk factors. Agreement between RBPT and i-ELISA was determined using a Kappa coefficient (*κ*). The overall animal-level seroprevalence was 25.9% based on i-ELISA. Logistic regression analysis revealed that odds of infection were significantly higher in females (OR = 1.8, 95% CI: 1.2–2.5, *p* = 0.002) and in young animals than in adults (OR = 3.6, CI: 2.1–6.2, *p* < 0.001). In addition, odds of infection were higher during the wet season (OR = 3.4, CI: 3.2–5.2, *p* < 0.001), in cattle reared in rural farms (OR = 4.8, CI: 2.0–11.5, *p* < 0.001), in cattle reared in areas, not in contact with wildlife (OR = 2.9, CI: 1.4–2.3, *p* = 0.004), and in medium-sized farms (OR = 12.5, CI: 6.9–22.9, *p* < 0.001). These findings confirm that bovine brucellosis was prevalent among commercial cattle farms in the ECZT, posing a serious public health concern to the community living in these settings. The one health approach should be adopted for effective control of brucellosis.

## 1. Introduction

Brucellosis is a zoonotic infectious disease caused by bacteria of the genus *Brucella*. Being zoonotic, the disease affects humans and a number of wild and domestic animals, including cattle. Brucellosis is highly contagious in animals and cross-species transmission of some *Brucella* species can occur [1]. Brucellosis in cattle (bovine brucellosis), also called contagious abortion or Bang's disease [2], is caused by *Brucella abortus* (*B*. *abortus*) and occasionally by *B*. *melitensi*s and *B*. *suis*.

In animals, the disease is characterized by late-term abortion, infertility, reduced milk, placenta retention, and secondary endometritis. Although a semi-intensive system is a common dairy management system in Tanzania, it is not uncommon to find dairy animals grazing close to or in communally owned land. This is due to scarcity of land because natural pasture is the most important source of feed. Higher brucellosis prevalence was previously reported in communally-grazed cattle compared to those reared under semi- or intensive production systems [3, 4]. In Tanzania, a bovine brucellosis seroprevalence ranging between 0.3% and 60.8% has been reported [5]. Brucellosis causes substantial economic losses. For example, a study in India reported the average estimated costs of treatment following a case of abortion, repeat breeding, and retention of placenta in cattle were USD 4, USD 5, and USD 7, respectively, [6].

Infected farm animals are the main source of human infections. Humans are infected through the consumption of infected meat, milk, and dairy products and by direct contact with infected animals through handling abortions, dystocia, and parturitions. Human brucellosis is popularly known as undulant fever, Crimean fever, Mediterranean fever, remitting fever, Maltese fever, goat fever, or Gibraltar fever [2]. Human brucellosis is debilitating and can end up in permanent injury and disability leading to financial loss attributable to medical expenses and loss of working hours [7].

Brucellosis affects not only the health of people in the poorest community but also their livelihood by reducing the productivity of their livestock. The control of the disease requires a thorough understanding of its epidemiology in different susceptible populations. The first report of human brucellosis in Tanzania was presented in 1935 [8].

Brucellosis is a disease of major socio-economic importance worldwide, particularly in low-income countries where disease control programs are either inadequate or nonexistent. The burden that the diseases impose on low-income countries has led the World Health Organization (WHO) to classify it as one of the world's leading “neglected” zoonotic diseases [9]. Brucellosis affects not only the health of people in the poorest communities but also their livelihoods by reducing the productivity of their livestock. Control of the disease requires a thorough understanding of its epidemiology in different susceptible populations.

The increase of human population in urban centers such as of the eastern coast zone of Tanzania (ECZT) has led to an expansion of commercial cattle farming especially in peri-urban areas to meet the increasing demand for milk and dairy products [10]. Moreover, growing awareness on nutritional needs and improved purchasing power of consumers has also scaled up commercial cattle farming. In attempts to meet the demand through increased production, the livestock sector also exposes consumers to public health concerns associated with production, products, and by-products. Among the concerns is brucellosis.

There is limited information on the magnitude of brucellosis in commercial cattle farms in the ECZT, although they contribute to a substantial amount of the milk supplied and consumed in urban areas. The lack of brucellosis control programs is particularly worrying in expanding dairy systems, where husbandry practices are known to favor disease spread [11].

Therefore, the purpose of this study was to estimate seroprevalence and establish risk factors of brucellosis in commercial cattle farms in selected districts of the ECZT in order to provide useful information for devising disease control programs in Tanzania.

## 2. Methods

### 2.1. Study Area

**Figure 1:**
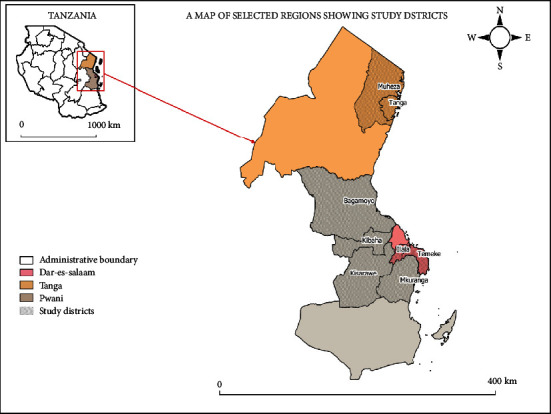
Map of the study area showing the districts selected for the study of the seroprevalence and risk factors of brucellosis in commercial cattle farms from selected districts of the eastern coast zone, Tanzania.

The study was conducted in the ECZT (Figure 1). The zone is characterized by a bimodal rain pattern (October to December and March to May) and in between is a dry spell. The zone was purposively selected because of its accessibility and the presence of a high milk supply chain which depends on commercial cattle farms as a source of milk to meet demands in this area, which has a relatively high population. Selected districts included Temeke and Ilala from the Dar es Salaam region; Mkuranga, Kibaha, Bagamoyo, and Kisarawe from the Pwani region and Muheza and Tanga city from the Tanga region. Twenty farms were purposively selected based on the accessibility and willingness of the farmers to participate in the study.

**Figure 2:**
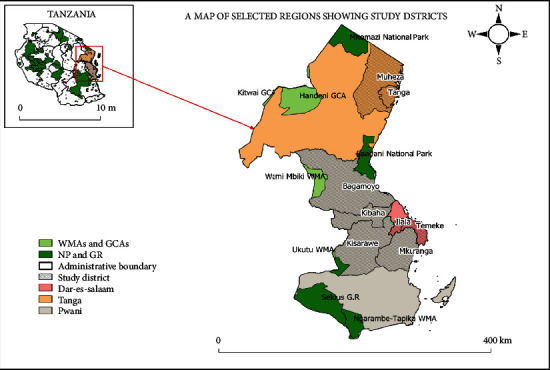
A map showing study districts in the eastern coast zone of Tanzania which are bordering national parks, forest reserves, or wildlife management areas. The districts were selected for the study of the seroprevalence and risk factors of brucellosis in commercial cattle farms.

Apart from having relatively large human populations, some of the districts in the study area are bordered by national parks, forest reserves, and wildlife management areas (Figure 2), and this could potentially play part in the epidemiology of brucellosis in such a setting. The districts which were bordering wildlife included Kisarawe, Mkuranga, and Bagamoyo, all from the Pwani region. In this study, Kibaha, Ilala, Temeke, and Tanga cities were considered as peri-urban, whereas Bagamoyo, Mkuranga, Kisarawe, and Muheza were considered as rural.

### 2.2. Study Design

This was a retrospective study which used 1,052 serum samples collected from 20 commercial cattle farms during a disease surveillance program by Tanzania Veterinary Laboratory Agency (TVLA) in Dar es Salaam, Tanzania. Samples were collected from October 2019 to April 2021. The samples were retrieved from the TVLA serum bank and subjected to the rose bengal plate test (RBPT) and indirect enzyme-linked immunosorbent assay (i-ELISA) to detect anti-*Brucella* antibodies.

### 2.3. Study Population

The target populations were cattle reared in commercial cattle farms, which in this study, were categorized as young (below one-year-old) and adults (one year and above). With regard to herd size, herds with less than 20 animals were considered as small; those with 20 to 100 cattle were considered as medium, whereas those with more than 100 cattle were regarded as large herds. All samples were collected from animals that had no history of brucellosis vaccination.

### 2.4. Sample Processing and Data Analysis

The laboratory work was carried out at TVLA, Temeke, Dar es Salaam. The testing procedure for RBPT was conducted as described by [12] as a screening test and then confirmed using i-ELISA. Data from laboratory analysis were entered, organized, and coded in Microsoft Excel 2016 spreadsheets, before being imported into STATA 14® (Stata Corp. College, Texas, USA) for analysis. Descriptive statistics were employed to determine the prevalence of the disease and other frequencies and proportions. Prevalence was based on the i-ELISA results and was presented in two levels, namely, individual (animal) level and farm (herd) level. A univariate analysis was conducted to assess the association between different factors and disease status. Examined factors included sex, age, herd size, season, farm location (peri-urban vs rural), and in contact vs not in contact with wildlife. Potential risk factors were selected for inclusion into a multivariate logistic regression modeling using a *p* value cut-off point of 0.1. A logistic regression modeling using a backward elimination method was used to determine risk factors using *p* > 0.05 as a criterion for removal from the model. Prior to building a final model, variables were tested for interaction effects using cross-product terms and for multiple-collinearity using the collinearity matrix index.

Agreement between RBPT and i-ELISA as diagnostic tests for bovine brucellosis was determined using Cohen's Kappa coefficient (*κ*). The Kappa value was interpreted as one of the following: poor (*κ* = 0), slight (0.01 < *κ* < 0.20), fair (0.21 < *κ* < 0.40), moderate (0.41 < *κ* < 0.60), almost perfect (0.61 <v*κ* < 0.80), and excellent (0.81 < *κ* < 1.00) [13].

For all analyses, a *p* value of less than 0.05 at a 95% confidence interval was considered statistically significant.

## 3. Results

### 3.1. Seroprevalence of Brucellosis at Animal-Level

Overall, animal-level seroprevalence was 25.9% (95% CI: 23.2–28.6%; *n*/*N* = 269/1052) and 29.9% (95% CI: 27.1–32.7%; *n*/*N* = 315/1052) by i-ELISA and RBPT, respectively.  Table 1 illustrates the seroprevalence of brucellosis at the animal-level (within farm), stratified by different factors. Prevalence ranged from 0 to 98.1%, with a mean of 26.7 ± 31.5%. Results showed that there was a significant association between all examined factors and brucellosis seropositivity (Table 1). Male cattle had significantly higher seroprevalence compared to females, while adult cattle had a significantly lower prevalence than young cattle. Tanga region had the highest seroprevalence, followed by Pwani, and Dar es salaam had the lowest. Higher prevalence was reported in rural farms than in peri-urban farms and during the wet season. Medium-sized farms had significantly higher prevalence, compared to large and small farms which had comparable prevalence. Farms that were bordering wildlife areas had lower prevalence compared to those not bordering wildlife areas.

### 3.2. Seroprevalence of Brucellosis at Farm Level

Overall farm-level seroprevalence was 70.0% (95% Confidence Interval, CI: 48–92%; *n*/*N* = 14/20) and 75.0% (95% CI: 54.2–98.5%; *n*/*N* = 15/20) based on i-ELISA and RBPT, respectively.  Table 2 shows the seroprevalence of brucellosis at the farm level, stratified by different factors. Herd size ranged from 6 to 173 with a mean of 53 ± 48.9. Seroprevalence was found to increase with herd size (43.0 to 100%) but the differences were not significant. Also, higher seroprevalence was observed in rural farms compared to peri-urban farms and those that were not close to wildlife game reserves or national parks compared to those that were in contact with wildlife. However, the differences were not significant.

### 3.3. Agreement between I-ELISA and RBPT Assays

Kappa statistic for assessment of agreement between i-ELISA and RBPT tests was estimated to be 0.89 (*Z* = 28.7, *p* ≤ 0.001) which implied an excellent agreement between the tests.

### 3.4. Risk Factors of Infection

On the univariate analysis, all examined factors were ruled as putative factors for brucellosis. These were sex, age, season, geographical location, contact with wildlife, and herd size. A multivariate logistic regression analysis identified all putative risk factors to be significant predictors of infection (Table 3). A logistic regression modellings was also used to investigate risk factors for brucellosis at the farm level and no factor was found to be significantly predicting the transmission of infection.

## 4. Discussion

This study confirmed that bovine brucellosis was prevalent among commercial cattle farms in six districts of the eastern coast zone of Tanzania. This signifies a serious public health threat to local populations, particularly those working in, or consuming raw dairy products from those exposed dairy farms. The overall farm-level prevalence was relatively high (70%) indicating a high spread of the disease across farms. The prevalence was comparable to the one reported in a study carried out in Zambia (62%) [14]. The animal-level seroprevalence was 25.9%, which was in line with the findings of studies done in Zimbabwe [15], Nigeria [16], and Ghana [17] which reported a prevalence of 30.1%, 24.0%, and 24.5%, respectively. However, the findings were contrary to other studies which reported a much lower prevalence of 2.4% as reported by Chota et al. [18] in Tanzania, 5.5% reported in South Africa [19], and 7.7% reported in Northern Malawi [20]. Furthermore, contrary to the findings of this study, higher animal-level prevalence of 40.1% and 45.9% were reported in Nigeria [11] and Angola [21], respectively. The variations in results observed in different studies may probably be attributed to several factors such as the sampling techniques, sample sizes, different diagnostic tests, and interpretations of results.

Sex was observed to be significantly associated with the occurrence of brucellosis in the study area. It was evident that female cattle were 1.8 times more likely to be seropositive as compared to male cattle. This observation was similar to other findings as articulated by Ferede et al. [22]; Degefu et al. [23]; Din [24] and Assenga et al. [25]. The lower seroprevalence of brucellosis in male animals could be attributed to the fact that the serological response of male animals to *Brucella* infection is limited [26]. As a result, it has been reported that infected male animals usually show low antibody titers [27].

In this study, the prevalence of brucellosis in cattle was significantly associated with animal age with higher odds of infection in young compared to adult cattle (OR = 3.6). Age has been referred to as one of the intrinsic factors associated with brucellosis [28]. The decrease of *Brucella* seropositivity with the age of animals contrasted with other studies which reported a higher risk of infection with increasing age [28–30]. However, it concurred with the findings from another study by Omer [31]. Several factors may account for the difference observed in this study. It is likely that in endemic areas the risk of *Brucella* infection (and thus seroconversion) is greater in younger animals as compared to older animals, some of which could be seronegative possibly due to latency, which is not uncommon in mature animals [16, 28]. Higher seropositivity in young animals can also be attributed to maternal antibodies which could still be in circulation when samples were taken. In addition, the arbitrary range of the age categories used in this study may have contributed to the observed results. Different results could have been observed with more age categories.

The proportions of seropositive animals differed significantly between the wet season and dry seasons. The wet season was found to be a brucellosis risk factor with the odds of seropositivity 3.4 times higher during the wet season as compared to the dry season. In the dry season, the feeding system of animals that is practiced by many intensive farming systems can serve as a potential risk factor but this is likely to play a role when fodder is collected from areas used by indigenous traditional cattle which encroach the peri-urban and urban settings [3]. The breeding cycle (parturition or abortions) in pastoral areas is often naturally synchronized with a wet season and feed availability, a condition which accelerates contamination and maintenance of the pathogens in the environment. In contrast, a lower likelihood of brucellosis during the dry season could probably be due to the lower survival rate of *Brucella* species in aborted materials in dry seasons and can also be explained by stall feeding that minimises contacts between herds and animals.

Geographical location was found to be related to the likelihood of being brucellosis positive in this study. Cattle from farms located in rural districts were 4.8 more likely to be brucellosis seropositive compared to cattle from peri-urban farms. This finding could probably be attributed to the fact that in rural settings, there is no restriction on animal movements [32]. It is documented that the dynamics and frequent relocation of pastoral herds may increase the likelihood of these herds coming into contact with herds in commercial farms and increase the likelihood of disease transmission [33].

In the current study, logistic regression models showed that animals from the districts (Bagamoyo, Kisarawe, and Mkuranga) that were bordering wildlife management areas, forest reserves and national parks were less likely to be infected. This finding that contact with wildlife was a “protective” factor seems to be contrary to many studies [25, 34, 35], which reported proximity to wildlife as a risk factor. However, all these studies seem to have worked on the indigenous breed of cattle that were kept extensively in the areas of their study. This finding could possibly be due to the type of breed. It has previously been observed that cross-bred cattle had relatively lower brucellosis seroprevalence compared to indigenous breeds [3, 36–38]. However, there seems to be disagreement between studies regarding breed susceptibility as other studies have reported higher susceptibility among cross-bred cattle [39, 40]. Hence, as data were retrospectively collected, contact with wildlife being a protective factor could be resulting from confounders; and further studies are needed to further elucidate this observation.

This study revealed that, generally, herd size was significantly associated with brucellosis seropositivity, particularly at the farm level. Farm-level seroprevalence increased with herd size with prevalence lowest in small herds (less than 20 cattle) and highest in large herds (more than 100 cattle). A number of studies have reported higher odds for seropositivity with increased herd size [10, 33, 39, 41, 42]. This could be associated with several factors, such as a higher number of animals tested in larger farms, which increases the probability of detecting at least one seropositive animal and/or the reason that as the number of animals goes up the likelihood of transmission of the disease by contact among them increases as well [43]. Although this bias could have been eliminated by a proper sampling procedure, given that this was a retrospective study, validity of the sampling procedure could not be ascertained.

At the animal level, seroprevalence was higher in the medium-sized herds compared to small and large herds. When comparing the odds of infection, odds were significantly higher in cattle from medium-sized herds as compared to those from small herds but did not differ between cattle in large herds and those in small herds. The reason for the higher seroprevalence of brucellosis in medium-sized herds could probably be attributed to the indiscriminative replacement of animals from infected herds or poor hygiene and management as compared to large-sized herds. This finding is similar to an observation made in a study by Deka et al. [44]. On the other hand, the lower prevalence of brucellosis in small-sized herds could be associated with the herd and/or farm management. Small-sized herds normally graze on pastures that are close to the farm, avoiding contact with other herds or utilization of common roads. Because premises for small herds are relatively smaller, cleaning, disinfection, and manure removal procedures are easier and less time-consuming to the farmer. Since large farms have been reported to be more susceptible to *Brucella* infection, large farms could possibly be owned by farmers who have more resources and are more knowledgeable and this may result in low disease prevalence. In this context wealth and education are confounding factors that prevent the association between large herd size and the occurrence of brucellosis.

With regard to serological test comparison, there was a substantial agreement observed between RBPT and i-ELISA (*k* = 0.89). These findings contradict with observations made in other studies conducted by Jaguar (2003) who reported a moderate agreement (*κ* = 0.44) and by Neha et al. [45] who reported a slight agreement (*κ* = 0.44) between the tests. The variation might be due to the lack of repeatability of the tests between laboratories and technicians.

One limitation of this study which can be acknowledged is the fact that a convenience sample was used. This may be associated with the risk that the group may not be a true representative of the population. Nevertheless, the sample enabled estimation of the disease status in the area.

## 5. Conclusion

In conclusion, this study confirms that *Brucella* infection circulates among commercial cattle farms supplying milk to urban consumers in the ECZT, posing a serious public health concern to the community living in this setting. Sex, age, season, geographical location, and farm size were found to be risk factors for the spread of the brucellosis among animals. The one health approach should be adopted by involving key stakeholders (public health workers, animal health worker, and policy makers) to reduce the brucellosis prevalence and effectively control brucellosis in the ECZT.

## Figures and Tables

**Table 1:** Animal-level seroprevalence of brucellosis stratified by different factors, determined from 1,052 serum samples collected from 20 commercial dairy cattle farms in the eastern coast zone of Tanzania.

Variable	Levels	No. tested	No. positive	Seroprevalence (%)	95% CI	*p* value
Sex	Male	332	60	18.1	13.9–22.3	<0.001
	Female	720	209	29.0	25.7–32.3	

Age	Adult (>1 year)	975	226	23.2	20.5–25.8	<0.001
	Young (<1 year)	77	43	55.8	44.7–66.9	

Region	Dar-es-Salaam	160	11	6.9	2.9–10.8	<0.001
	Pwani	530	113	21.3	17.8–24.8	
	Tanga	362	145	40.1	34.9–45.1	

Location	Rural	422	142	33.7	29.1–38.1	<0.001
	Peri-urban	630	127	20.2	17.0–23.3	

Season	Dry	786	133	16.9	14.3–19.5	<0.001
	Wet	266	136	51.1	45.1–57.1	

Herd size	Large	442	27	6.1	3.9–8.3	<0.001
	Medium	539	237	43.9	39.8–48.2	
	Small	71	5	7.0	0.9–13.1	

Contact with wildlife	No	530	156	29.4	18.1–25.2	0.004
	Yes	522	113	21.6	25.5–33	

**Table 2:** Farm-level seroprevalence of brucellosis reported in 20 commercial dairy farms in selected districts of the eastern coast zone of Tanzania.

Variable	Levels	No. tested	No. positive	Seroprevalence (%)	*p* value
Herd size	Large	3	3	100.0	0.121
	Medium	10	8	80.0	
	Small	7	3	43.0	

Location	Peri-urban	11	7	63.6	0.492
	Rural	9	7	77.8	

Contact with wildlife	No	12	8	66.7	0.690
	Yes	8	6	75.0	

**Table 3:** Multivariate logistic regression analysis showing risk factors associated with the brucellosis seroprevalence in cattle from 20 commercial dairy farms in the eastern coast zone of Tanzania.

Variable	Levels	Odds ratio	95% CI	*p* value
Sex	Male^a^			
	Female	1.8	1.2–2.8	0.006

Age	Adult^a^			
	Young	3.6	2.1–6.2	<0.001

Season	Dry^a^			
	Wet	3.4	2.2–5.2	<0.001

Location	Peri-urban^a^			
	Rural	4.8	2.0–11.5	<0.001

Contact with wildlife	Yes^a^			
	No	2.97	1.4–2.3	0.004

Herd size	Small^a^			
	Medium	12.5	6.9–22.9	<0.001

^a^ = Reference category. CI=Confidence interval.

## Data Availability

All data pertaining to the current study are available from the corresponding author upon a reasonable request.
